# Short-term thermal acclimation of dark respiration is greater in non-photosynthetic than in photosynthetic tissues

**DOI:** 10.1093/aobpla/plz064

**Published:** 2019-10-02

**Authors:** Nicholas G Smith, Guoyong Li, Jeffrey S Dukes

**Affiliations:** 1 Department of Biological Sciences, Texas Tech University, Lubbock, TX, USA; 2 Department of Forestry and Natural Resources, Purdue University, West Lafayette, IN, USA; 3 Department of Biological Sciences, Purdue University, West Lafayette, IN, USA; 4 Purdue Climate Change Research Center, Purdue University, West Lafayette, IN, USA; 5 International Joint Research Laboratory for Global Change Ecology, School of Life Sciences, Henan University, Kaifeng, China

**Keywords:** Carbon cycling, climate change, *R*_d_, respiratory demand, terrestrial biosphere models, warming

## Abstract

Thermal acclimation of plant respiration is highly relevant to climate projections; when included in models, it reduces the future rate of atmospheric CO_2_ rise. Although all living plant tissues respire, few studies have examined differences in acclimation among tissues, and leaf responses have received greater attention than stems and roots. Here, we examine the short-term temperature acclimation of leaf, stem and root respiration within individuals of eight disparate species acclimated to five temperatures, ranging from 15 to 35 °C. To assess acclimation, we measured instantaneous tissue temperature response curves (14–50 °C) on each individual following a 7-day acclimation period. In leaves and photosynthetic stems, the acclimation temperature had little effect on the instantaneous tissue temperature response of respiration, indicating little to no thermal acclimation in these tissues. However, respiration did acclimate in non-photosynthetic tissues; respiratory rates measured at the acclimation temperature were similar across the different acclimation temperatures. Respiratory demand of photosynthetic tissue increased with acclimation temperature as a result of increased photosynthetic demands, resulting in rates measured at the acclimation temperature that increased with increasing acclimation temperature. In non-photosynthetic tissue, the homeostatic response of respiration suggests that acclimation temperature had little influence on respiratory demand. Our results indicate that respiratory temperature acclimation differs by tissue type and that this difference is the consequence of the coupling between photosynthesis and respiration in photosynthetic, but not non-photosynthetic tissue. These insights provide an avenue for improving the representation of respiratory temperature acclimation in large-scale models.

## Teaser

Our study found that temperature acclimation of respiration differs by tissue type (i.e. leaves, stems and roots). Tissue that does not photosynthesize was found to have more homeostatic responses to temperature than photosynthetic tissue. This was found due to the strong linkage between photosynthetic biochemistry and respiration fluxes. These results suggest that plant respiratory responses to changing temperatures, such as future warming, will be tissue type-specific. The link to photosynthesis found in our study provides an avenue for improving the representation of these responses in carbon cycle models.

## Introduction

Respiratory carbon release from the land surface is one of the largest fluxes of carbon dioxide (CO_2_) between the atmosphere and the Earth’s surface. Respiration by plants makes up about half of this flux (Ciais *et al.* 2013). As a result, terrestrial biosphere models are highly sensitive to the representation of plant respiratory processes, including respiratory thermal acclimation (Atkin *et al.* 2008; Booth *et al.* 2012; Slot *et al.* 2014; Lombardozzi *et al.* 2015; Smith *et al.* 2016; Huntingford *et al.* 2017), a process that may become increasingly important as climates warm globally (Ciais *et al.* 2013). While thermal acclimation of plant respiration has been observed in a variety of studies, it is still not often included in terrestrial biosphere model simulations (Smith and Dukes 2013), likely due to a poor mechanistic understanding of the response (Atkin *et al.* 2005).

Thermal acclimation of respiration is defined as a change in the instantaneous response of respiration to temperature as a result of a longer-term change in temperature (Atkin and Tjoelker 2003; Atkin *et al.* 2005; Smith and Dukes 2013). This commonly results in some combination of a decrease in the slope of the relationship between respiration and temperature and/or a reduction in respiratory rates measured at a common temperature (Atkin and Tjoelker 2003). This effect can dampen respiratory responses to temperature. As such, respiratory rates measured at a tissue temperature identical to the temperature to which the tissue is acclimated are relatively homeostatic across acclimation temperatures (Loveys *et al.* 2003; Slot and Kitajima 2015). These acclimation responses tend to be stronger in tissues developed at the new temperature or acclimated to the new temperature for a longer period of time (Atkin and Tjoelker 2003)

Acclimation responses likely depend, in part, on changes in maintenance demand that result from changes in temperature (Lambers *et al.* 1983; Amthor 1984). For instance, increases in temperature may result in increased maintenance demand to support functioning of non-respiratory enzymes, reducing the degree of respiratory acclimation observed in response to short-term changes in temperature. In support of this, studies (e.g. Loveys *et al.* 2003) have found that tissues developed at a new temperature show stronger respiratory acclimation than ones that developed before a change in acclimation temperature (Atkin *et al.* 2005) and respiratory acclimation in leaves tends to increase with time following the transfer to a new temperature regime (Slot and Kitajima 2015). This suggests that higher maintenance requirements in tissues developed before a temperature change may limit the degree of respiratory acclimation, particularly in leaves. Still, it is unclear how these mechanisms may play out in other plant tissues, such as stems and roots.

Respiratory thermal acclimation may differ by tissue type; however, this effect has received little attention in the literature (Atkin and Tjoelker 2003; Smith and Dukes 2013) and terrestrial biosphere models typically simulate stem and root respiration simply as a function of leaf respiration (Atkin *et al.* 2017). Leaves (e.g. Slot and Kitajima 2015), stems (e.g. Maseyk *et al.* 2008) and roots (e.g. Jarvi and Burton 2013) have each been observed to acclimate to changes in temperature in previous studies. Nonetheless, comparisons of acclimation among tissue types are rare.

For leaves and photosynthetic stems, acclimation is likely tied to temperature effects on photosynthetic biochemistry (Gifford 2003; Smith and Dukes 2017). Indeed, previous controlled environment (Smith and Dukes 2017) and space-for-time substitution (Atkin *et al.* 2015) studies have found that the ratio of leaf dark respiration to photosynthetic capacity is similar under different acclimation temperatures. Notably, Smith and Dukes (2017) showed non-homeostatic leaf respiratory responses to five acclimation temperatures in 11 species, an effect that coincided with increases in photosynthetic capacity.

In non-photosynthetic tissues, such as woody stems and roots, one would not expect respiration to be closely linked to photosynthetic processes. Instead, demand for respiratory products in these tissues is more likely to be related to growth and transport (Tjoelker *et al.* 1999; Covey-Crump *et al.* 2002; Atkin *et al.* 2007). If these processes are not influenced by changes in acclimation temperature, one might expect a greater degree of observed acclimation in non-photosynthetic tissues (e.g. as seen by Loveys *et al.* 2003). Additionally, spectral differences among tissues as well as insultation of roots by soil may result in non-uniform changes in tissue temperatures resulting from a change in air temperature. As acclimation is a response to tissue, not air, temperature, these differences could result in differential acclimation responses among tissues.

In one of the only studies that has examined respiratory temperature acclimation across multiple tissues, Loveys *et al.* (2003) found greater acclimation of roots than leaves in previously developed, but not newly developed leaves. The authors attributed this effect to the rapid growth and turnover of the roots, such that some portion of the measured roots had developed under the new temperatures (Loveys *et al.* 2003). However, this may have also been due to an increased demand for respiratory products in leaves due to higher rates of photosynthetic processes, but no change in demand in roots. Stem tissue was not compared to root and leaf tissue. In general, the dearth of studies examining acclimation differences among plant tissues limits our ability to understand and predict how respiratory fluxes will be influenced by temperature.

Here, we examine respiratory thermal acclimation of leaves, stems and roots in response to a short-term (i.e. 7-day) change in temperature in eight species (*Betula alleghaniensis*, *Cucumis sativa*, *Glycine max*, *Pinus nigra*, *Pinus pinaster*, *Pinus pinea*, *Pinus sylvestris* and *Zea mays*). We assessed short-term thermal responses to mimic the types of changes that plants may experience over intra-annual timescales and because photosynthesis is known to acclimate over short time periods (Veres and Williams 1984; Battaglia *et al.* 1996; Atkin *et al.* 2000; Turnbull *et al.* 2002; Gunderson *et al.* 2010). We used a variety of plant species in order to make our results more generalizable across taxa, but did not have reason to expect species to differ in their responses. Individual plants were acclimated to one of five temperatures from 15 to 35 °C and the instantaneous response of respiration to temperature was measured for each tissue from 14 to 50 °C. We hypothesized that acclimation to warmer temperatures would reduce the instantaneous tissue temperature sensitivity of respiration for each tissue. We expected this reduced sensitivity to result in more homeostatic respiratory rates at the acclimation temperatures than would be expected from the instantaneous responses alone. We expected this acclimation to be greater in non-photosynthetic than in photosynthetic tissues because these tissues are not influenced by changes in photosynthetic processes that result from changes in acclimation temperature.

## Methods

### Growth conditions

We used species that varied in growth form, including trees (*B. alleghaniensis*, *P. nigra*, *P. pinaster*, *P. pinea*, *P. sylvestris*) and crops that included herbaceous species (*C. sativa*, *G. max*) and a grass (*Z. mays*) (Table 1). The individuals were grown from seed in a 50 %/50 % mixture of field soil (sandy loam; pH: 6.9) and potting soil (Sungro Metro Mix 510; Sungro Horticulture, Agawam, MA, USA) in 1.9-L pots. Plants were not pot-bound by the end of the experiment. Individuals were germinated and grown for an initial period in controlled environment glass houses at ~25 °C. Relative humidity inside the glass house was 58 % on average over the course of this growing period. The glasshouse was sprayed with reflective paint to reduce the risk of overheating. This also acted to reduce photosynthetically active radiation (PAR). As such, PAR was supplemented using 400-Watt overhead lights, with daily PAR reaching a maximum of ~1500 µmol m^−2^ s^−1^. Overhead lights were set to a constant 16/8-h light/dark schedule. Individuals were watered when soil became dry. Individuals were provided fertilizer (Miracle Gro 24-8-16 N-P-K; Scotts Company LLC, Marysville, OH, USA) following initial germination and about every 60 days thereafter to avoid nutrient limitation.

**Table 1.** Number of individuals sampled per species per acclimation temperature for dark respiration and, in parentheses, dark respiration and photosynthesis. *T*_a_ = acclimation temperature. *Stem respiration was not measured for *Z. mays*.

	*T*_a_ = 15 °C	*T*_a_ = 20 °C	*T*_a_ = 25 °C	*T*_a_ = 30 °C	*T*_a_ = 35 °C	Average
Betula alleghaniensis	1 (1)	3 (3)	0 (0)	3 (3)	1 (1)	1.6 (1.6)
Cucumis sativa	3 (3)	2 (2)	1 (1)	1 (1)	2 (2)	1.8 (1.8)
Glycine max	5 (2)	2 (0)	1 (1)	6 (6)	1 (1)	3 (2)
Pinus nigra	2 (2)	3 (3)	3 (2)	2 (2)	2 (2)	2.4 (2.2)
Pinus pinaster	1 (1)	1 (1)	3 (1)	2 (2)	4 (4)	2.2 (1.8)
Pinus pinea	1 (1)	1 (1)	2 (2)	1 (1)	0 (0)	1 (1)
Pinus sylvestris	1 (1)	1 (1)	2 (2)	3 (3)	3 (3)	2 (2)
Zea mays*	3 (3)	2 (2)	2 (1)	4 (0)	3 (0)	2.8 (1.2)
Average	2.1 (1.8)	1.9 (1.6)	1.8 (1.3)	2.8 (2.3)	2 (1.6)	2.1 (1.7)

### Acclimation treatments

The time between germination and transfer to growth chambers differed by species. For trees, this period was ~6 months. As such, all trees were juveniles and ranged from ~30 to 50 cm in height. For annual species, this time period was ~1–2 months. In all cases, species were transferred before the production of reproductive tissues.

After germination in the glass houses and the initial growth period, individuals were transferred to environmentally controlled growth chambers (Conviron E15; Controlled Environments Inc., North Branch, MN, USA) for a 7-day acclimation period. Chambers were set to either 15, 20, 25, 30 or 35 °C (acclimation temperature or *T*_a_; see Table 1 for the number of individuals of each species acclimated to each temperature). Relative humidity was set to 50 %, lights were set to a 16/8-h light/dark schedule with lights increasing in intensity (25 % every 15 min) during the first hour and decreasing in intensity (25 % every 15 min) during the last hour of the 16-h light period. Photosynthetically active radiation was ~1470 µmol m^−2^ s^−1^ during peak hours inside the chamber. Plants in the chamber were provided water when soil became dry. Each individual was acclimated to only one of the five acclimation temperatures.

### Gas exchange measurements

Following the 7-day acclimation treatment, instantaneous temperature response curves were developed for leaf (*R*_d,leaf_), stem (*R*_d,stem_) and root (*R*_d,root_) dark respiration (*R*_d_). To build these curves, *R*_d_ measurements were taken at tissue temperatures of 14, 23, 32, 41 and ~50 °C using two LiCor 6400 portable photosynthesis systems running simultaneously (LiCor Biosciences, Lincoln, NE, USA) with a standard 3 cm × 2 cm chamber and attached light source turned off. The cuvette was sealed with putty to ensure there were no leaks. The lack of leaks was confirmed by leak tests (i.e. blowing on the chamber) during each measurement. All measurements proceeded inside the chamber following a 1-h dark adaptation period prior to the first measurement and during which the entire plant was placed in a dark growth chamber. The chamber was kept dark throughout the course of the measurements. Both the cuvette and growth chamber temperatures were adjusted to alter tissue temperatures. Measurements were made successively at progressively warmer temperatures from 14 to ~50 °C. Full temperature responses of leaves, then stems and then roots were recorded. For root measurements, roots were carefully removed from soil to reduce breakage and measured while attached to the plant. Tissue temperatures were measured using the internal thermocouple on the LiCor 6400, ensuring that tissues were in contact with the thermocouple during measurement. The warmest tissue temperature was set to the maximum temperature attainable by the machine and thus varied by individual (mean ± SE: leaf = 44.49 ± 0.013 °C, stem = 45.36 ± 0.012 °C, root = 45.14 ± 0.011 °C). For each temperature setting, we took 30 measurements over 30 s after matching the infrared gas analyzers. The average of these was used for analysis. One individual per portable photosynthesis system was measured each day for a total of two individuals measured per day. Note that *R*_d,stem_ was not measured for *Z. mays* due to the thickness of the stems. *R*_d,stem_ was measured for all other species.

Prior to, but on the same day as, all respiratory measurements, net photosynthesis (*A*_net_) by intercellular CO_2_ (*C*_i_) curves was taken on each individual. To build these curves, *A*/*C*_i_ measurements were taken at leaf temperatures of 14, 23, 32, 41 and ~50 °C using the LiCor 6400 portable photosynthesis instruments (LiCor Biosciences, Lincoln, NE, USA). Both cuvette and growth chamber temperatures were adjusted to alter leaf temperatures. Responses were measured first at the temperature at which the plant was grown. This measurement was made to ensure stomata were open and responding to changes in CO_2_ and was discarded prior to analysis. Measurements were then made successively at progressively warmer temperatures from 14 to ~50 °C. Leaf temperatures were measured using the internal thermocouple on the LiCor 6400. The warmest leaf temperature was set to the maximum temperature attainable by the machine and thus varied by individual (mean ± SE: 44.31 ± 0.10 °C). Light inside the chamber was set to a saturating rate of 1200 µmol m^−2^ s^−1^. Humidity inside the leaf chamber was maintained at ~60 %, but was occasionally lower at high temperatures. In those cases, water was added to the flow path by adding water (<5 mL) to the soda lime to achieve the highest level of humidity possible. *A*/*C*_i_ curves were generated using leaf chamber CO_2_ values of (in order): 400, 300, 200, 100, 50, 400, 400, 600, 800, 1000, 1200, 1500 and 2000 µmol mol^−1^ CO_2_ for C_3_ species and 400, 300, 200, 100, 50, 0, 400, 400, 600 and 800 µmol mol^−1^ CO_2_ for C_4_ species. Photosynthetic parameters, including the maximum rate of Rubisco carboxylation (*V*_cmax_), the maximum rate of electron transport for Ribulose-1,5-bisphosphate regeneration (*J*_max_) and, for C_4_ species, the maximum rate of phosphoenol pyruvate carboxylation (*V*_pmax_), were fit for each *A*/*C*_i_ curve using the ‘plantecophys’ package in R (Duursma 2015), following Smith and Dukes (2017). For all gas exchange measurements, we ensured that leaf fluxes were at steady state before beginning measurements.

Following gas exchange analyses, tissue inside the chamber was removed and fresh tissue projected area was assessed using a scanner and ImageJ (Schneider *et al.* 2012); if necessary, gas exchange rates were adjusted accordingly. Tissues were then dried to a constant mass at 65 °C. Fluxes were converted to a dry mass basis (i.e. µmol CO_2_ g^−1^ s^−1^).

### Temperature response curve fitting

The temperature responses of leaf, stem and root *R*_d_ for each individual plant were fit using a third-order polynomial described by O’Sullivan *et al.* (2013), as in Smith and Dukes (2017):

$$\begin{array}{l} R_{T}=\exp(a+bT_{l}+cT_{l}^{2}) \end{array}$$ (1)

where *R*_T_ (µmol g^−1^ s^−1^) is the process rate at the leaf temperature *T*_l_, *a* corresponds to the exponential rate of *R*_T_ at 0 °C (µmol g^−1^ s^−1^), *b* is a parameter describing the change in rates with temperature at temperatures near 0 °C and *c* is a parameter describing the change in this increase with increasing temperature. The average root mean squared error (RMSE) for the *R*_d_ temperature response curves of leaves, stems and roots was 0.09, 0.11 and 0.16 µmol g^−1^ s^−1^, respectively.

As a proxy for demand for the workings of photosynthetic machinery, the temperature response of *V*_cmax_ was also fit using equation 1 for each plant. The average RMSE was 0.13 µmol m^2^ s^−1^. Following fitting, *V*_cmax_ data were converted to a per-gram trate (i.e. µmol g^−1^ s^−1^) to aid in comparison to *R*_d_ data.

### Data analysis

To test whether the instantaneous response of *R*_d_ differed as a result of changes in *T*_a_, we used mixed-model analyses of covariance with the ‘lmer’ function in the ‘lme4’ package (Bates *et al.* 2015) in R version 3.5.0 (R Development Core Team 2019). The temperature response parameters *a*, *b* and *c* for *R*_d_ were used as dependent variables. The species (categorical variable), 7-day temperature to which the individual was acclimated (*T*_a_; i.e. 15, 20, 25, 30 or 35 °C; continuous variable), tissue type (leaf, photosynthetic stem, non-photosynthetic or root; categorical variable) and the interaction between tissue type and *T*_a_ were included as predictor variables in each model. Individual was included as a random factor in the models. To explicitly examine the influence of photosynthetic processes on *R*_d_ of each tissue we calculated rates of *R*_d_ of each tissue and *V*_cmax_ at tissue temperatures equal to *T*_a_ (*R*_d,acc_ and *V*_cmax,acc_). We then calculated the ratio of each tissue’s *R*_d,acc_ to *V*_cmax,acc_ (*R*_d,acc_/*V*_cmax,acc_) and fit a similar mixed-effects model as above. Following model fitting, significance testing was performed by calculating the Wald χ ^2^ for each model parameter using the ‘Anova’ function in the ‘car’ package (Fox and Weisberg 2011). For all models, we visually examined residual plots following model fitting to ensure that necessary assumptions for model comparisons were met (Zuur *et al.* 2009).

*Post hoc* analyses were done using the ‘emmeans’ package (Lenth 2018). Specifically, *post hoc* least squared mean slope and intercept values describing the relationship between the response variables and *T*_a_ were calculated using the fitted model for each tissue type. This allowed for these calculations to account for all independent variables fit in the model. We examined whether slopes from these models were different from 0 for each tissue type using a *t*-statistic test with degrees of freedom calculated using Kenward-Roger approximation. This was done using the ‘test.emmGrid’ function in the ‘emmeans’ package (Lenth 2018). We used planned contrasts to compare slopes of photosynthetic versus non-photosynthetic tissues to the acclimation temperatures following our original hypothesis. This was done using *t*-ratio tests using the ‘contrast’ function in the ‘emmeans’ package (Lenth 2018).

Using the least squared mean slope and intercept values for the relationship between the response variables and *T*_a_, we were able to calculate fitted *R*_d_ values at different tissue temperatures (*T*_t_). Specifically, we calculated *R*_d_ at *T*_a_ = *T*_t_. To assess the degree of acclimation for each, we used the ‘Homeostasis Method’ (Loveys *et al.* 2003). This involved calculating the ratio of *R*_d_ at *T*_a_ = *T*_t_ to *R*_d,25_ at *T*_a_ = 25 °C for plants acclimated to 15, 20, 30 and 35 °C, which we refer to *Acclim*_Homeo_. For ease of comparison, *R*_d,25_ at *T*_a_ = 25 °C was used in the denominator when calculating *Acclim*_Homeo_ for plants acclimated to 15 and 20 °C and *R*_d,25_ at *T*_a_ = 25 °C was used in the numerator when calculating *Acclim*_Homeo_ for plants acclimated to 30 and 35 °C. As such, in all cases, *Acclim*_Homeo_ values of 1 indicate homeostatic *R*_d_ rates, with values further from 1 indicating progressively less homeostasis. We used plants acclimated to 25 °C as the reference because this was the temperature at which the plants were germinated and grown prior to being placed in the growth chamber.

Due to poor germination, death and equipment malfunctioning, the number of individuals per species per acclimation temperature differed. On average 2.1 individuals per species per acclimation temperature were measured. This sample size was the result of a choice to maximize the number of species used to assess the generality of our hypotheses, which were not species-specific, but rather tissue-specific. To match this hypothesis, we included acclimation temperature as a continuous variable in our models and did not include any interaction terms with species. Thus, the low number of species per acclimation temperature was not an issue for our models. Table 1 shows the number of individuals per species per acclimation temperature used for the analysis. The mixed-model analyses of variance used here are robust for handling unbalanced designs (Zuur *et al.* 2009).

All data used for the analyses described here can be found at https://github.com/SmithEcophysLab/tissue_respiration (doi: 10.5281/zenodo3445384).

## Results

### The basal rate of dark respiration (*a*)

**Figure 1.**
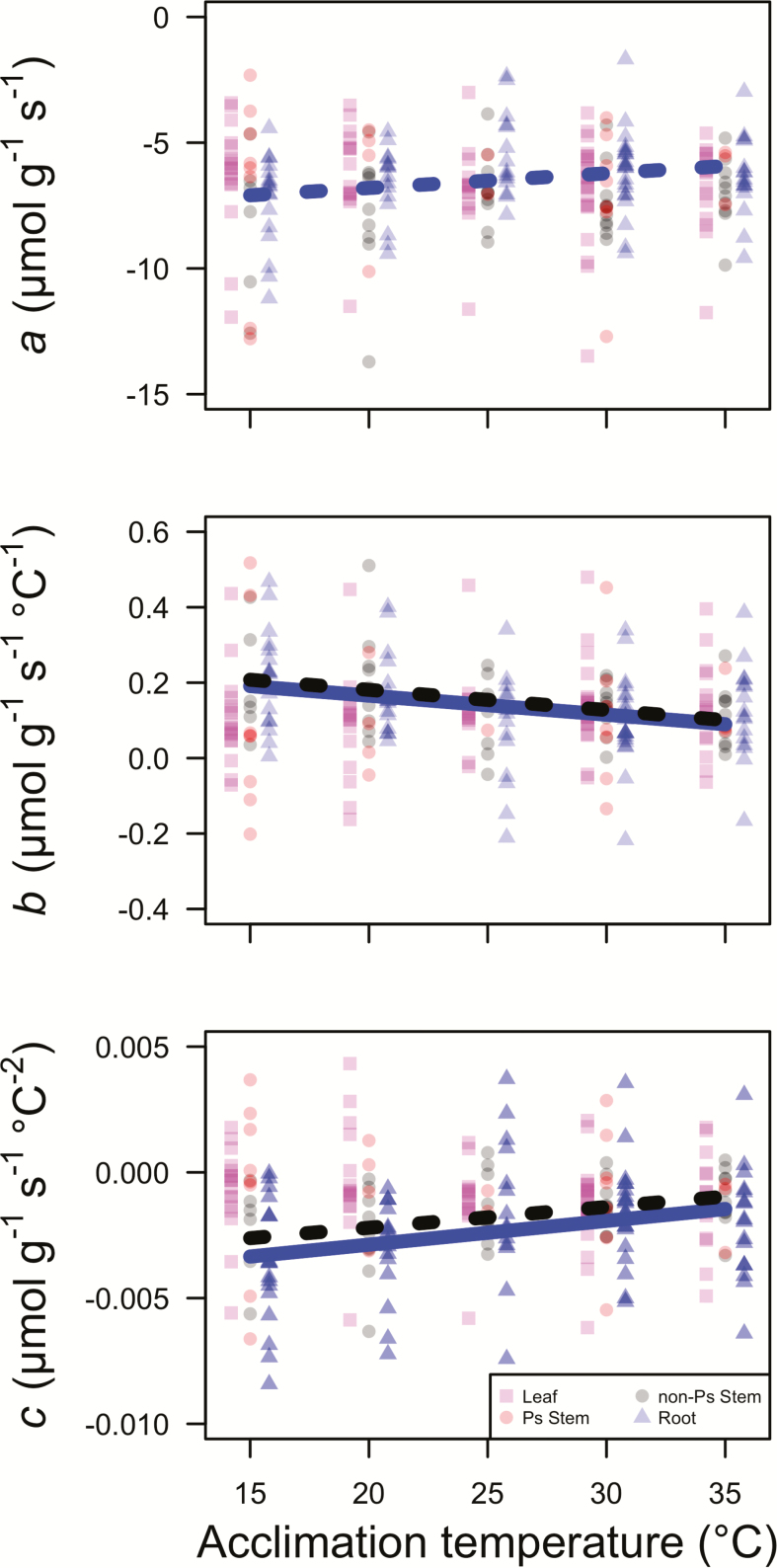
The effect of acclimation temperature (*T*_a_) on parameter values describing the instantaneous temperature response of dark respiration (*R*_d_). Leaf, photosynthetic (Ps) stem, non-Ps stem and root parameters are indicated by pink squares, red circles, grey circles and blue triangles, respectively. Leaf and root points are jittered along the *x*-axis by −0.8 and 0.8 °C, respectively, to improve visibility. Significant (*P* < 0.05) and marginally significant (*P* < 0.10) slopes are shown with solid and dashed lines, respectively, with colours corresponding to tissue type (i.e. black = non-Ps stem, blue = root). Slope values are least squared means from the mixed-model analyses of variance.

The parameter, *a*, that describes the rate of *R*_d_ at a tissue temperature of 0 °C was not detectably influenced by tissue type, the temperature at which the plants were acclimated (acclimation temperature; *T*_a_), or the interaction between the two factors (*P* > 0.05 in all cases; Table 2). *Post hoc* analyses of slopes did indicate a marginally significant increase in *a* rates with *T*_a_ for roots (*P* = 0.054; Table 3; Fig. 1), but no effect for other tissue types (*P* > 0.10 in all cases; Table 3; Fig. 1). A planned contrast found no difference between slopes of the *a*–*T*_a_ relationship between photosynthetic and non-photosynthetic tissue (*t*_213_ = 1.79, *P* > 0.05). The *a* value did differ by species (*P* < 0.05; Table 2). *Post hoc* comparisons indicated that the only statistically different (*P* < 0.05) species combination was between the species with the lowest *a* rates (*P. pinaster*; estimated marginal mean *a* across tissue types = −7.57 µmol g^−1^ s^−1^) and the species with the highest *a* rates (*Z. mays*; estimated marginal mean *a* across tissue types = −5.90 µmol g^−1^ s^−1^). All other species had statistically similar *a* rates.

**Table 2.** Results from mixed-model analysis of variance testing thermal acclimation of instantaneous temperature response parameters across tissue types*. *P values less than 0.05 are indicated in bold. *T*_a_ = acclimation temperature, *a* corresponds to the exponential rate of *R*_T_ at 0 °C (µmol g^−1^ s^−1^), *b* is a parameter describing the change in rates with temperature at temperatures near 0 °C and *c* is a parameter describing the change in this increase with increasing temperature, *R*_d,acc_/*V*_cmax,acc_ is the ratio of dark respiration to the maximum rate of Rubisco carboxylation at tissue temperatures equal to the acclimation temperature.

		*a*		*b*		*c*		*R*_d,acc_/*V*_cmax,acc_	
	df	χ ^2^	*P*	χ ^2^	*P*	χ ^2^	*P*	χ ^2^	*P*
Species	7	17.65	**0.014**	9.97	0.190	10.93	0.142	40.02	**<0.001**
*T*_a_	1	1.27	0.259	2.01	0.156	2.32	0.128	14.95	**<0.001**
Tissue	3	3.37	0.338	3.71	0.295	26.57	**<0.001**	30.18	**<0.001**
*T*_a_ × Tissue	3	5.38	0.146	9.68	**0.021**	12.08	**0.007**	13.03	**0.004**

**Table 3.** Slopes of the response of the instantaneous temperature response parameters to *T*_a_*. *Slope indicates the least squared mean slope of the relationship between the parameter (i.e. *a*, *b* or *c*) and *T*_a_. The SE is the standard error of the least squared mean slope. Degrees of freedom (df) were estimated using Kenward-Roger approximation. The *t*-ratio test examined whether the slopes were significantly different from 0. Values with *P* values less than 0.05 and 0.1 are indicated in bold and italics, respectively.

		*a*					*b*					*c*				
Tissue	Photosynthetic	Slope	SE	df	*t*-ratio	*P*	Slope	SE	df	*t*-ratio	*P*	Slope	SE	df	*t*-ratio	*P*
Leaf	Yes	−0.027	0.030	219.7	−0.91	0.361	0.002	0.002	221.9	1.17	0.244	−0.000041	0.000032	221.9	−1.29	0.200
Stem	Yes	0.002	0.053	222.9	0.04	0.968	0.001	0.004	222.6	0.42	0.675	−0.000033	0.000056	222.6	−0.59	0.555
Stem	No	0.060	0.043	222.7	1.38	0.168	−0.005	0.003	222.7	−1.87	*0.062*	0.000083	0.000046	222.7	1.82	*0.071*
Root	No	0.058	0.030	219.7	1.94	*0.054*	−0.005	0.002	221.9	−2.56	**0.011**	0.000094	0.000032	221.9	2.99	**0.003**

### The thermal response of the instantaneous rate of dark respiration near 0 °C (*b*)

There was an interaction between tissue type and *T*_a_ for the parameter, *b*, that describes the instantaneous response of *R*_d_ to temperature near a tissue temperature of 0 °C (*P* < 0.05; Table 2). *Post hoc* analyses of slopes indicated that root *b* decreased with *T*_a_ (*P* < 0.05; Table 3; Fig. 1), and that non-photosynthetic stem *b* similarly decreased with *T*_a_, but this response was only marginally significant (*P* = 0.062; Table 3; Fig. 1). The *post hoc* slope analysis indicated that leaf and photosynthetic stem *b* were not significantly influenced by *T*_a_ (*P* > 0.10; Table 3; Fig. 1). These results suggested that non-photosynthetic tissue *b* was more responsive to *T*_a_ than photosynthetic tissue *b*, an effect confirmed by a planned contrast showing that slopes of the relationship between *b* and *T*_a_ differed between photosynthetic and non-photosynthetic tissue (*t*_216_ = −2.67, *P* < 0.01). The *b* parameter did not differ by species (*P* > 0.10; Table 2).

### The change in the instantaneous thermal response of dark respiration with increasing temperature (*c*)

There was a significant interaction between tissue type and *T*_a_ on the parameter, *c*, that describes the change in the instantaneous thermal response of *R*_d_ with increasing tissue temperature (*P* < 0.01; Table 2). *Post hoc* analyses of slopes indicated that root *c* increased with *T*_a_ (*P* < 0.01; Table 3; Fig. 1) and that non-photosynthetic stem *c* similarly increased with *T*_a_, but this effect was only marginally significant (*P* = 0.071; Table 3; Fig. 1). Leaf and photosynthetic stem *c* were not influenced by *T*_a_ (*P* > 0.10; Table 3; Fig. 1). These results suggest that non-photosynthetic tissue *c* was more responsive to *T*_a_ than photosynthetic tissue *c*, an effect confirmed by a planned contrast showing that slopes of the relationship between *c* and *T*_a_ differed between photosynthetic and non-photosynthetic tissue (*t*_216_ = 2.96; *P* < 0.01). The *c* parameter did not differ by species (*P* > 0.10; Table 2).

### Modelled thermal acclimation of dark respiration

**Figure 2.**
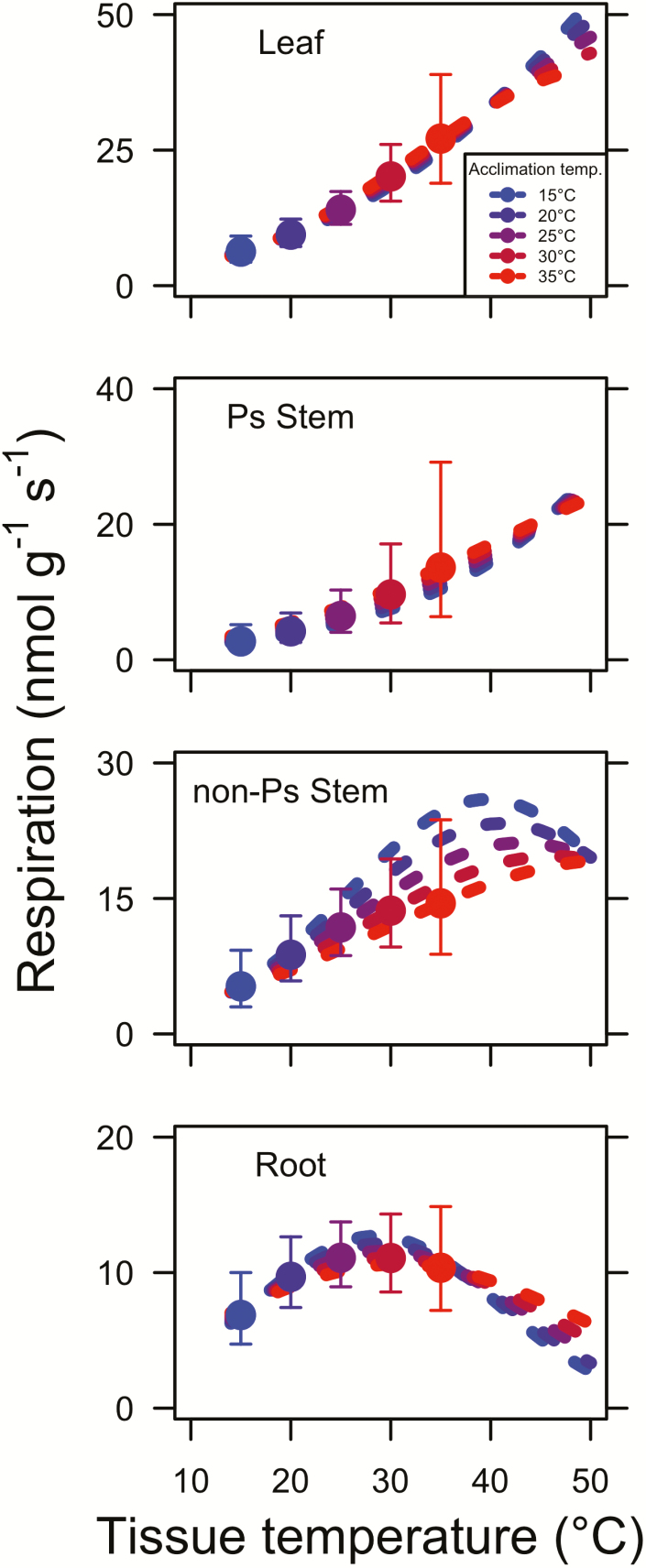
The instantaneous temperature response of leaf, photosynthetic (Ps) stem, non-photosynthetic stem and root respiration. Instantaneous curves were created using the parameters obtained in Table 3. Points indicate least squared mean (±SE) values for respiration rates measured at leaf temperatures *T*_a_ equal to the tissue temperature. Throughout, blue, blue-purple, purple, red-purple and red points and lines indicate values for plants acclimated to 15, 20, 25, 30 and 35 °C, respectively (see inset legend in top panel).

We assessed thermal acclimation of dark respiration for each tissue type by modelling the instantaneous response for each *T*_a_ assessed in the study (i.e. 15, 20, 25, 30 and 35 °C). We did this using the least squared mean slope and intercept values for the relationship between parameters *a*, *b* and *c* and *T*_a_ from the mixed-model analysis of variance (Table 3) to calculate parameter values at *T*_a_ values of 15, 20, 25, 30 and 35 °C. We also calculated modelled respiration rates at *T*_a_ equal to the tissue temperature. These calculations showed that instantaneous responses to tissue temperature were strongest in photosynthetic tissues, leaves in particular, and dampened in non-photosynthetic tissue, roots in particular (Fig. 2).

### Homeostasis of dark respiration under varying temperatures

This difference in thermal acclimation between photosynthetic and non-photosynthetic tissue was also apparent in *Acclim*_Homeo_ values (Table 4). Values closer to 1 indicate a greater degree of homeostasis in respiration rates. Leaves and photosynthetic stems had average *Acclim*_Homeo_ values of 0.58 and 0.55, respectively, while root and non-photosynthetic stems had values of 0.89 and 0.71, respectively (Table 4). This effect was driven by greater homeostasis at warmer temperatures in non-photosynthetic tissue. In fact, across all tissue types, *Acclim*_Homeo_ tended to be furthest from 1 in plants acclimated to 15 °C (average = 0.48; Table 4). This suggests that low acclimation temperatures tended to reduce *R*_d_ regardless of tissue type.

**Table 4.** Calculated homeostasis (*Acclim*_Homeo_) values for each tissue at each acclimation temperature (*T*_a_)*. *All values are in relation to *R*_d_ values at *T*_a_ = 25 °C. Values closer to 1 indicate a greater degree of homeostatic acclimation. *T*_a_ = acclimation temperature; Ps = photosynthetic.

*T*_a_	Leaf	Ps stem	Non-Ps stem	Root	Average
15 °C	0.45	0.42	0.45	0.62	0.48
20 °C	0.67	0.65	0.74	0.87	0.73
30 °C	0.70	0.67	0.86	1.00	0.81
35 °C	0.52	0.47	0.82	1.07	0.72
Average	0.58	0.55	0.72	0.89	0.68

### The ratio of dark respiration to photosynthetic capacity

**Figure 3.**
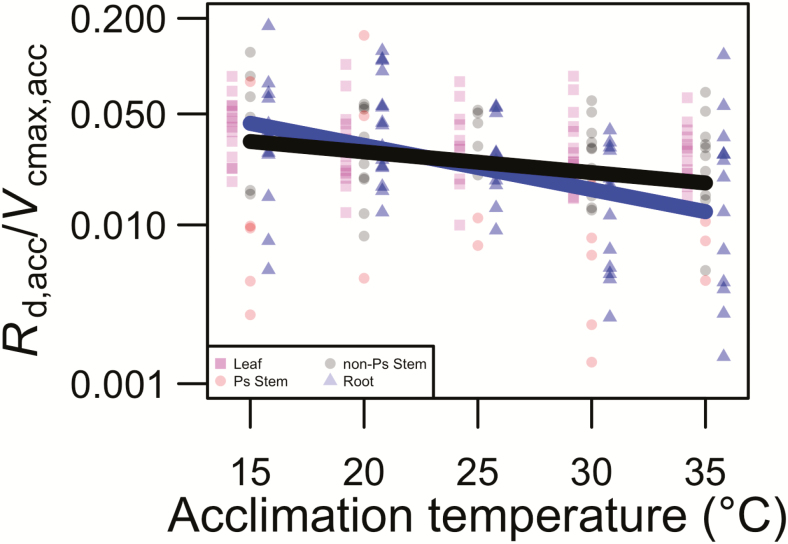
The effect of acclimation temperature (*T*_a_) on the ratio of dark respiration to the maximum rate of Rubisco carboxylation at tissue temperature equal to the acclimation temperature (*R*_d,acc_/*V*_cmax,acc_) for each tissue type leaf, photosynthetic (Ps) stem, non-Ps stem and root values are indicated by pink squares, red circles, grey circles and blue triangles, respectively. Leaf and root points are jittered along the *x*-axis by −0.8 and 0.8 °C, respectively, to improve visibility. Significant (*P* < 0.05) slopes are shown with solid lines, with colours corresponding to tissue type (i.e. black = non-Ps stem, blue = root). Slope values are least squared means from the mixed-model analyses of variance. The line equations are *y* = e^−0.063*x* − 2.20^ and *y* = e^−0.030*x* – 2.94^ for black (i.e. non-photosynthetic stem) and blue (i.e. root) lines, respectively. Data are plotted on a log scale.

The ratio of dark respiration to the maximum rate of Rubisco carboxylation at the tissue temperature equal to *T*_a_ (*R*_d,acc_/*V*_cmax,acc_) depended on *T*_a_ in some tissue types, but not others (tissue type × *T*_a_; *P* < 0.01; Table 2). *Post hoc* analyses of slopes indicated that root and non-photosynthetic stem *R*_d,acc_/*V*_cmax,acc_ decreased with *T*_a_ (*P* < 0.05 in both cases; Fig. 3), but that neither leaf nor photosynthetic stem *R*_d,acc_/*V*_cmax,acc_ was influenced by *T*_a_ (*P* > 0.05 in both cases; Fig. 3). These results suggest that non-photosynthetic tissue *R*_d,acc_/*V*_cmax,acc_ was more responsive to *T*_a_ than photosynthetic tissue *R*_d,acc_/*V*_cmax,acc_. This conclusion was supported by a planned contrast showing that slopes of the relationship between *R*_d,acc_/*V*_cmax,acc_ and *T*_a_ differed between photosynthetic and non-photosynthetic tissue (*t*_154_ = −2.00; *P* < 0.05). The *R*_d,acc_/*V*_cmax,acc_ varied by species (*P* < 0.01; Table 2). *Post hoc* comparisons found that the only species that differed significantly in *R*_d,acc_/*V*_cmax,acc_ (*P* < 0.05) was that with the highest ratio (*P. pinea*; estimated marginal mean *R*_d,acc_/*V*_cmax,acc_ across tissue types = 0.041) and that with lowest ratio (*Z. mays*; estimated marginal mean *R*_d,acc_/*V*_cmax,acc_ across tissue types = 0.010).

## Discussion

Here, we asked: As is commonly assumed by large-scale models (e.g. Oleson *et al.* 2013), is thermal acclimation of dark respiration (*R*_d_) similar across tissue types (leaves, stems and roots)? And, if not, then why do tissue types differ? Using eight species across four diverse plant functional types, we found that thermal acclimation in response to a short acclimation period (7 days) was apparent in non-photosynthetic tissues, but was not observed in photosynthetic tissues. This pattern is consistent with the results found by Loveys *et al.* (2003), who found *Acclim*_Homeo_ ratios of leaves and roots that were nearly equivalent to the results found here. Our stem results provide further insight into the mechanisms driving this response and suggest that photosynthetic tissues have reduced short-term thermal down-regulation of dark respiration, thus decreasing *Acclim*_Homeo_ values. Photosynthetic data taken on the same individuals showed that increases in *T*_a_ resulted in an increase in maximum rates of Rubisco carboxylation (*V*_cmax_) and electron transport (*J*_max_) (Smith and Dukes 2017). This effect likely increased respiratory demand for photosynthetic processes, which may have limited any respiratory down-regulation.

Past work has shown that dark respiration acclimation of leaves is primarily driven by acclimation of *V*_cmax_ (Wang *et al.* 2018). Indeed, *V*_cmax_ has been used as a proxy to model leaf dark respiration for decades (Farquhar *et al.* 1980; Collatz *et al.* 1991). Our results support the idea that dark respiration of photosynthetic tissue is highly correlated to *V*_cmax_, as evidenced by the fact that *R*_d,acc_/*V*_cmax,acc_ ratios were similar across all acclimation temperature treatments in photosynthetic tissues. As such, *V*_cmax_ may be a suitable proxy for simulating dark respiration thermal acclimation of photosynthetic tissue in large-scale models.

However, our results suggest that dark respiration is less sensitive to changes in acclimation temperature in non-photosynthetic tissue than in photosynthetic tissue. This was particularly true for acclimation temperatures between 20 and 35 °C. At these temperatures, rates of dark respiration were relatively homeostatic (*Acclim*_Homeo_ ratios ranging from 0.74 to 1.07). This may have been because growth and maintenance demands remained similar across these temperatures in these tissues, coupled with reduced respiratory temperature limitation at these temperatures. Indeed, at 15 °C *Acclim*_Homeo_ values tended to drop in all tissues, indicating that this low acclimation temperature may have induced some degree of temperature limitation to dark respiration.

The strong acclimation responses of non-photosynthetic tissue are consistent with previous stem and root respiratory thermal acclimation studies (e.g. Maseyk *et al.* 2008; Jarvi and Burton 2013). Our results do not fully clarify the drivers of the observed response. Our results do, however, suggest that non-photosynthetic tissue respiration cannot be modelled using photosynthetic tissue responses, as is commonly done (e.g. Oleson *et al.* 2013), as this approach may overestimate non-photosynthetic tissue respiration at high temperatures. Further work is necessary to understand the mechanisms driving temperature responses of non-photosynthetic dark respiration in order to reliably simulate this process in models.

Our results may have been related to the length of our acclimation time period. We chose a 7-day acclimation period to simulate short-term (i.e. intra-annual) variation in temperature that these plants might experience in the field. Slot and Kitajima (2015) used a meta-analysis to examine the *R*_d_ thermal acclimation of leaves, and found that acclimation increased with increasing duration of the experimental treatment. Thus, the leaves (and potentially other tissues) in our experiment may not have had time to fully adjust to the changed conditions. Additionally, the lack of leaf dark respiration acclimation observed in the photosynthetic tissue in our study contrasts with strong acclimation responses seen in longer-term studies of leaves (Heskel *et al.* 2016; Reich *et al.* 2016). However, this does not explain the acclimation seen by non-photosynthetic tissue in our study and, indeed, those studies did not report data on photosynthetic capacity (e.g. *V*_cmax_), which may have helped to explain the respiration acclimation observed. Further studies that examine the timescale of respiratory temperature acclimation across multiple tissue types would support a more refined representation of acclimation in large-scale models.

While the *b* and *c* parameters defining the shape of the temperature response curve did not differ by species, the *a* parameter defining the basal rate did show species specificity. This indicates that the species in this study did not show variation in the shape of the acclimation response, but did vary in the magnitude of their respiratory rates. These results were not surprising given previous reports of wide variation in basal respiration rates among species (e.g. Reich *et al.* 2007; Atkin *et al.* 2015; Heskel *et al.* 2016; Smith and Dukes 2018). The goal of our study was not to determine differences across species, but rather to use multiple species to broadly examine tissue-specific acclimation responses, which was reflected in the design of our statistical models in that species by *T*_a_ interactions were not included. Nonetheless, the low number of species used here was a limitation of our study and future work should build upon these results and examine whether tissue-specific temperature acclimation varies by species or plant type.

Taken as a whole, our results support the idea that respiration processes in models need to be timescale- and tissue-dependent. While we provide the data necessary to parameterize such statistical models (https://github.com/SmithEcophysLab/tissue_respiration), we suggest that these data instead be used to develop and test more mechanistic models of plant *R*_d_. Our results, coupled with those from previous studies mentioned above, suggest some core principles acting to drive respiration responses and acclimation to temperature. First, respiratory processes, under many conditions, are likely driven by demand for respiratory products. Respiration in plants acts to support processes such as enzyme turnover, carbohydrate export and growth (Amthor 1984) and many models, at least at the leaf scale, are already designed based on this principle and are capable of acting at timescales longer than instantaneous (i.e. they include acclimation) (Atkin *et al.* 2017). This mechanism could be extended to non-photosynthetic tissues, which, as we show in this study, are likely to acclimate differently than photosynthetic tissues.

## Sources of Funding

N.G.S. acknowledges support from Texas Tech University. This work was supported by a NASA Earth and Space Science Fellowship (NNX13AN65H) and a Purdue Climate Change Research Center Graduate Fellowship to N.G.S. J.S.D.’s contributions were supported in part by Hatch project 1000026 of the United States Department of Agriculture’s National Institute of Food and Agriculture. G.L. acknowledges funding from the National Natural Science Foundation of China (31270564).

## Contributions by the Authors

N.G.S., G.L. and J.S.D. designed the study. N.G.S. and G.L. carried out the research and performed the analyses. N.G.S., G.L. and J.S.D. wrote the manuscript.

## Conflict of Interest

None declared.

## Data Accessibility

All data and analysis scripts can be accessed at https://github.com/SmithEcophysLab/tissue_respiration (doi: 10.5281/zenodo3445384).
